# Improving the DSM-5 approach to cognitive impairment: Developmental prosopagnosia reveals the need for tailored diagnoses

**DOI:** 10.3758/s13428-024-02459-4

**Published:** 2024-07-08

**Authors:** Edwin J. Burns

**Affiliations:** Department of Psychology, Swansea University, Swansea, UK

**Keywords:** Diagnosis, Neurocognitive disorders, Prosopagnosia, Single case analysis, Mild cognitive impairment, Major, Subjective cognitive impairment, MCI, Transdiagnostic

## Abstract

The Diagnostic Statistical Manual of Mental Disorders (DSM-5) recommends diagnosing neurocognitive disorders (i.e., cognitive impairment) when a patient scores beyond – 1 SD below neurotypical norms on two tests. I review how this approach will fail due to cognitive tests’ power limitations, validity issues, imperfect reliabilities, and biases, before summarizing their resulting negative consequences. As a proof of concept, I use developmental prosopagnosia, a condition characterized by difficulties recognizing faces, to show the DSM-5 only diagnoses 62–70% (*n1* = 61, *n2* = 165) versus 100% (*n1* = 61) through symptoms alone. Pooling the DSM-5 missed cases confirmed the presence of group-level impairments on objective tests, which were further evidenced through meta-analyses, thus validating their highly atypical symptoms. These findings support a paradigm shift towards bespoke diagnostic approaches for distinct cognitive impairments, including a symptom-based method when validated effective. I reject dogmatic adherence to the DSM-5 approach to neurocognitive disorders, and underscore the importance of a data driven, transdiagnostic approach to understanding patients’ subjective cognitive impairments. This will ultimately benefit patients, their families, clinicians, and scientific progress.

## Introduction

The Diagnostic and Statistical Manual for Mental Disorders fifth edition (DSM-5, APA, 2013) is considered the gold standard guidance for practitioners diagnosing mental disorders in the United States. One section of the DSM-5 focuses on neurocognitive disorders which can be graded as reflecting mild and major cognitive impairment due to a variety of causes, including Alzheimer’s disease, Parkinson’s disease, HIV, and traumatic brain injury (Sachdev et al., 2014). Impairments are typically characterized by a reduction in cognitive or behavioral functioning within, or across, six key domains, covering perceptual-motor function, language, learning and memory, social cognition, complex attention, and executive function (Sachdev et al., 2014). To diagnose impairment, the DSM-5 developers recommend that a patient must score more poorly than one standard deviation below a neurotypical mean on two cognitive or behavioral tasks (Sachdev et al., 2014).

It could be claimed that the DSM-5 has been helpful in providing formal, and straightforward, standardized guidance to diagnose neurocognitive disorders. However, this approach has been criticized partly because the liberal criterion of – 1 SDs below a neurotypical mean will result in mistaken diagnoses (Schultz, 2010, 2013; Wakefield, 2013), i.e., 16% of the normally distributed general population would be diagnosed as abnormal on a single test, despite such individuals being cognitively intact. Thus, the DSM-5 in its current form poses risks to neurotypical patients by diagnosing impairments where none exist.

However, we must not ignore the converse risks absolute cut-offs pose when patients are erroneously rejected as cognitively intact, i.e., missed diagnoses. Such false negatives are rarely highlighted in critiques of the DSM-5 approach to neurocognitive disorders (Schultz, 2010, 2013; Wakefield, 2013), despite missed diagnoses and pathologizing normality often having a common cause, i.e., when patients’ diagnostic test performance distributions substantially overlap with neurotypicals. Numerous conditions associated with cognitive impairments suffer problems with missed diagnoses, including Long COVID (Costas-Carrera et al., 2022; Pihlaja et al., 2023) and dementia (Beishon et al., 2019; Potts et al., 2022), where patients will often appear, at least from their cognitive test results, indistinguishable from those absent of disease. It is therefore important for clinicians and researchers to understand the risks associated with missed diagnoses, and how they can occur. This will help avoid dogmatic thinking that the DSM-5 approach is infallible when assessing whether a patient is impaired.

### Can developmental prosopagnosia reveal the DSM-5’s limitations?

To illustrate how the DSM-5 approach can result in missed diagnoses, I present the case of developmental prosopagnosia (DP). This is a lifelong condition characterized by severe difficulties recognizing facial identity (Avidan & Behrmann, 2008; Bate et al., 2014; Behrmann & Avidan, 2005; Behrmann et al., 2005; Bennetts et al., 2024; De Haan, 1999; De Haan & Campbell, 1991; Duchaine & Nakayama, 2006; Halder et al., 2024; Maw et al., 2024; McConachie, 1976; Thomas et al., 2009), affecting 1.88– 6% of the general population (Burns, 2023; Burns et al., 2023; Gray et al., 2017; Kennerknecht et al., 2006, 2008a, 2008b). It can have a substantial negative impact upon peoples’ interpersonal, romantic and professional relationships, causing fear, anxiety and low self-confidence (Dalrymple et al., 2014; Yardley et al., 2008). While the causes of DP are unclear, it does run in families suggesting a possible genetic component (De Haan, 1999; Duchaine, Germine, et al., 2007; Grueter et al., 2007; Kennerknecht et al., 2008a, 2008b; Lee et al., 2010), which may account for the wide range of neural atypicalities they exhibit (Behrmann et al., 2007; Behrmann & Plaut, 2013; Burns et al., 2013, 2014; Fisher et al., 2016, 2017, 2020; Fox et al., 2011; Furl et al., 2011; Jiahui et al., 2018; Lohse et al., 2016; Manippa et al., 2023; Righart & de Gelder, 2007; Rivolta et al., 2014; Rosenthal et al., 2017; Song et al., 2015; Thomas et al., 2009; Towler et al., 2012, 2016, 2018; Van den Stock et al., 2008).

I chose this group for the current paper first, because I have experience working with them and the tests used to assess their problems. Second, these individuals suffer an extremely high proportion of potentially missed diagnoses (i.e., up to 85%) when using a cut-off of – 2 SDs on two cognitive tasks of face processing (Bate, Bennetts, Gregory, et al., 2019; Burns et al., 2023; Lowes et al., 2024). Owing to this, I had an a priori hypothesis (see Burns et al., 2023) that problems in diagnosing would remain, even if we had used the more liberal DSM-5 criteria for neurocognitive disorders.

While neurodevelopmental conditions like DP would not be included in the umbrella term of neurocognitive disorders by the DSM-5 developers (Sachdev et al., 2014), researchers have applied its principles to DP (DeGutis et al., 2023; Stumps et al., 2020). Similarly, even though the updated DSM-5 stresses that clinicians must not rigidly follow its recommended diagnostic cut-offs (DSM-5-TR Neurocognitive Disorders Supplement, APA, 2022), this is what some researchers have suggested the field adopt (DeGutis et al., 2023). Moreover, an argument could be made that acquired prosopagnosia, typically onsetting after an observable brain injury (Barton et al., 2001; Behrmann & Plaut, 2014; Bornstein & Kidron, 1959; Bukach et al., 2006; Bukach et al., 2012; Bukach et al., 2008; de Gelder & Rouw, 2000; Humphreys et al., 2007; Marotta et al., 2001), *would* come under the umbrella term of neurocognitive disorders. Importantly, this form suffers similar problems as DP whereby cases can perform too well on face processing tasks (Burns et al., 2023; Fysh & Ramon, 2022; Josephs & Josephs, 2024). Given acquired cases are exceptionally difficult to recruit in large numbers, and similar cognitive task-based diagnostic issues are present in both groups, it seems reasonable to use DP in the present paper to demonstrate the limitations of the DSM-5 approach to diagnosing.

### Reasons for missed diagnoses

In this section, I present reasons why developmental prosopagnosia may go undiagnosed through the DSM-5, building on prior work (Burns et al., 2023; Epihova & Astle, 2024; Lowes et al., 2024; McIntosh & Rittmo, 2021; Volfart & Rossion, 2024). My intention is to provide a basis upon which professionals dealing with other neurocognitive disorders can reflect on, and scrutinize, the potential issues with cognitive and behavioral tests they employ. This should encourage the adoption of a more accurate, data-driven approach for diagnosing and treatment, where researchers and clinicians recognize the limitations of the DSM-5 method, i.e., we should attempt to validate self-reported complaints with objective data, rather than using arbitrary cognitive task cutoffs to reject subjective complaints.

Before I begin, I should mention some researchers believe self-identified DP cases do not have the condition when their individual cognitive test scores miss diagnostic cutoffs. While theoretically possible, I largely reject this hypothesis. In my experience, historically missed cases describe qualitatively similar face recognition failures during interview as those who do meet criteria, and exhibit quantitatively comparable symptoms (Burns et al., 2023). Moreover, it is arguably easy to detect when you fail to recognize a familiar person during a conversation, as it is patently obvious that they know who you are, but you do not recollect them (Burns, 2023; Burns et al., 2023; Tsantani et al., 2021). Consider the types of conversations you have with familiar people; they are intuitively different from those struck up by strangers. As I suspect most, if not all, missed cases have DP, this review focuses on why the DSM-5 fails to diagnose those with the condition.

This first reason why the DSM-5 fails is because researchers and clinicians do not follow the guidance set out by those who develop diagnostic tests. This has been a consistent issue in the DP literature since the Cambridge Face Memory Test (CFMT), an unfamiliar face memory task widely used to diagnose developmental prosopagnosia, first came into print (Duchaine & Nakayama, 2006). Its developers reported that it failed to detect impairments at the – 2 SD level in 25% of DP cases, and 12.5% at – 1 SD. As a result, the authors stated that professionals should not solely rely on it for a diagnosis (Duchaine & Nakayama, 2006). Despite this, researchers and clinicians have not heeded these warnings. If you read the literature over the last 10–15 years, you will find impairment on the CFMT was essential for a diagnosis in the majority of papers (Burns et al., 2023; DeGutis et al., 2023), and I must admit to being guilty of this myself (Burns, Bennetts et al. 2017; Burns, Martin et al. 2017; Burns et al., 2014; Wilcockson et al., 2020). Thus, even when developers of diagnostic tests highlight their limitations, professionals will fail to acknowledge them. This will result in patients erroneously told they do not have developmental prosopagnosia, simply because the tests and cutoffs we enforce do not capture every patient’s impairment.

Why do tasks like the CFMT fail to detect atypicality in every self-identified case? One reason may be that such tests suffer imperfect ecological validity (Burns et al., 2023; Ramon et al., 2019). This occurs if they fail to fully capture the problems a patient experiences in the real world. Alternatively, such tasks may accurately reflect the problems they suffer from, but fail to clearly detect the superior abilities of neurotypicals. In either case, the performance distributions of DP and neurotypicals will overlap to such an extent that they render the DSM-5’s –1 SD cutoff ineffective for diagnostic purposes.

To illustrate how tests can potentially lack validity, let us consider the defining characteristics of DP: consistent failures when attempting to recognize personally familiar people, such as co-workers, friends and even family members. In an ideal world, it would seem sensible to use these people in our diagnostic tests. However, this is exceptionally impractical due to the consent requirements of all involved, and the time constraints on researchers and clinicians who must create such tasks. As a solution, a Famous Faces Test (FFT) is almost always used to aid a diagnosis (Bate & Tree, 2017; Burns et al., 2023; Dalrymple & Palermo, 2016), where patients are required to recognize images of highly familiar celebrities.

**Fig. 1:**
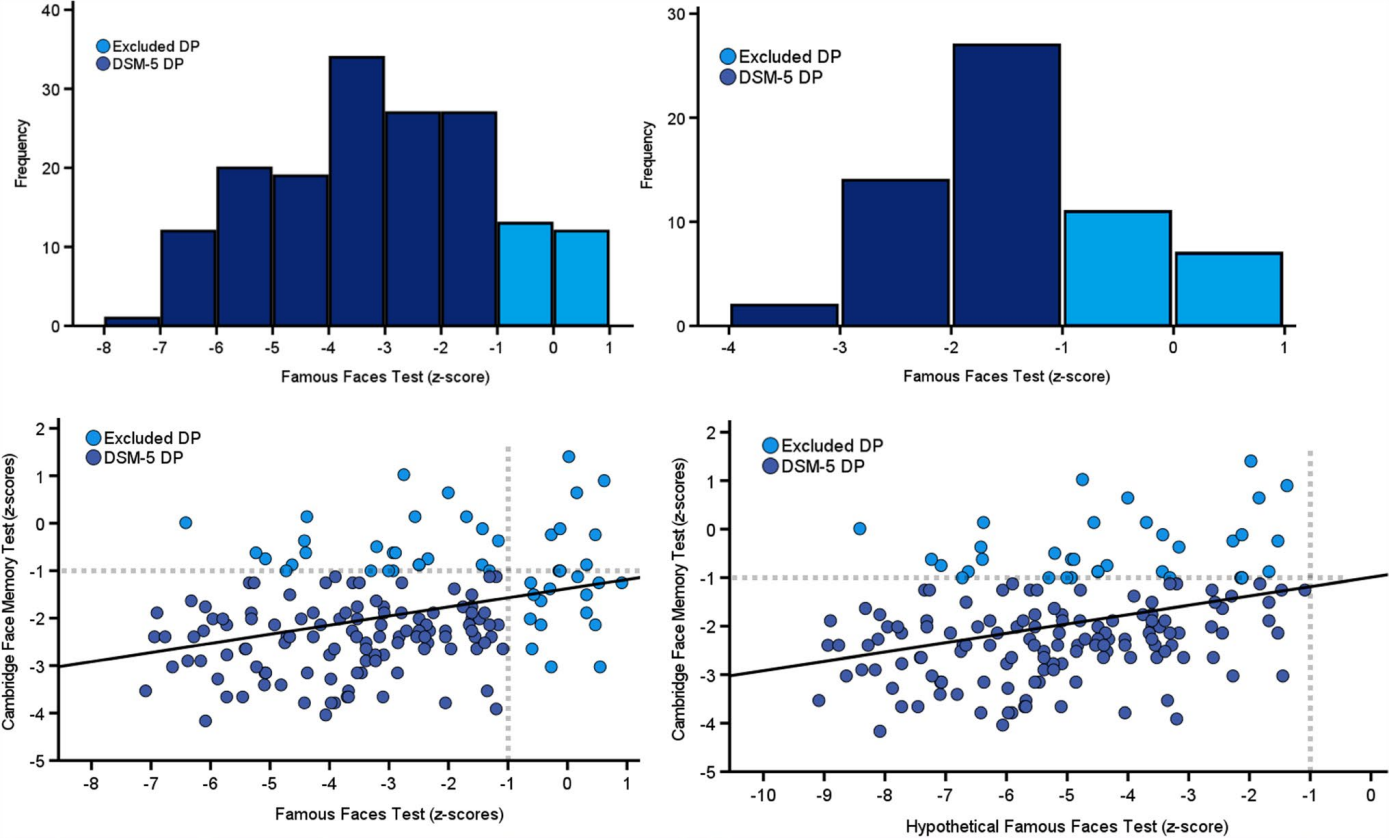
The *top left* and *right* panels demonstrate the – 1 SD cutoff removes the top end (*light blue*) of the DP FFT performance distribution, with 15% (*top left*: Bate, Bennetts, Gregory et al., 2019) and 30% (*top right*: Burns et al.,  2023) failing to meet criteria. In the *bottom left panel*, using Bate, Bennetts, Gregory et al. (2019) data, the – 1 SD cutoff on FFT (*x*-axis) has power to detect impairment in 85% of cases, while CFMT (*y*-axis) has 79% power. Requiring deficits on both means power can never be greater than the weakest of the two, with only 70% of self-identified DP cases (*dark blue*) meeting DSM-5 criteria and 30% excluded (*light blue*). In the *bottom right panel*, we present a hypothetical Famous Faces Test that has perfect sensitivity, i.e., 100% power at the – 1 SD level. Unfortunately, as we require impairment on a second imperfect test that has 79% power (i.e., the CFMT), we will only ever diagnose at this rate. This is despite some of the DP cases scoring almost – 9 SDs on the FFT. Please note, I simply subtracted – 2 from the Bate, Bennetts, Gregory et al. (2019) Famous Faces Test data to create this hypothetical data

However, this test cannot be easily standardized given that people of different cultures, different personal interests, and different age groups will be more familiar with certain famous faces than others. This may partly explain the heterogeneity of 15–35% of those who self-identify as suffering from developmental prosopagnosia failing to score below – 1 SD on this task (Bate, Bennetts, Gregory et al., 2019, Burns et al., 2023; Lowes et al., 2024). When employed, this cutoff simply removes the top end of the homogenous DP performance distribution when plotted with those who do meet criteria (Fig. 1). If we assume that they are all part of the same DP group as the distributions suggest, then the – 1 SD cutoff will inevitably exclude many from a diagnosis.

Why might people with DP score above the DSM-5 cutoff on the Famous Faces Test? These tasks almost always use a single, still image of each celebrity. Maybe neurotypicals rely more heavily on movement when recognizing familiar faces in the real world than DP cases. This means neurotypical performance when using photographs will be shifted down so that many DP cases land above the – 1 SD cutoff, despite their problems in real life. Another reason may be that people with developmental prosopagnosia have more problems in real-world settings than is captured by a computer screen-based Famous Faces Test^1^. This could occur if the brain processes celebrities, or rather celebrity photographs, to some extent differently from personal acquaintances (Ramon & Gobbini, 2018; Taylor et al. 2009; although see Herzmann et al., 2004; Wiese et al., 2022), where the recognition of the former may be partly intact in DP, while the latter is not. In any case, given that 15–35% of people with self-identified prosopagnosia perform too well on Famous Faces Tests (Bate, Bennetts, Gregory et al., 2019, Burns et al., 2023; Lowes et al., 2024), we must acknowledge such tasks’ validity limitations can theoretically cause missed diagnoses.

**Fig. 2:**
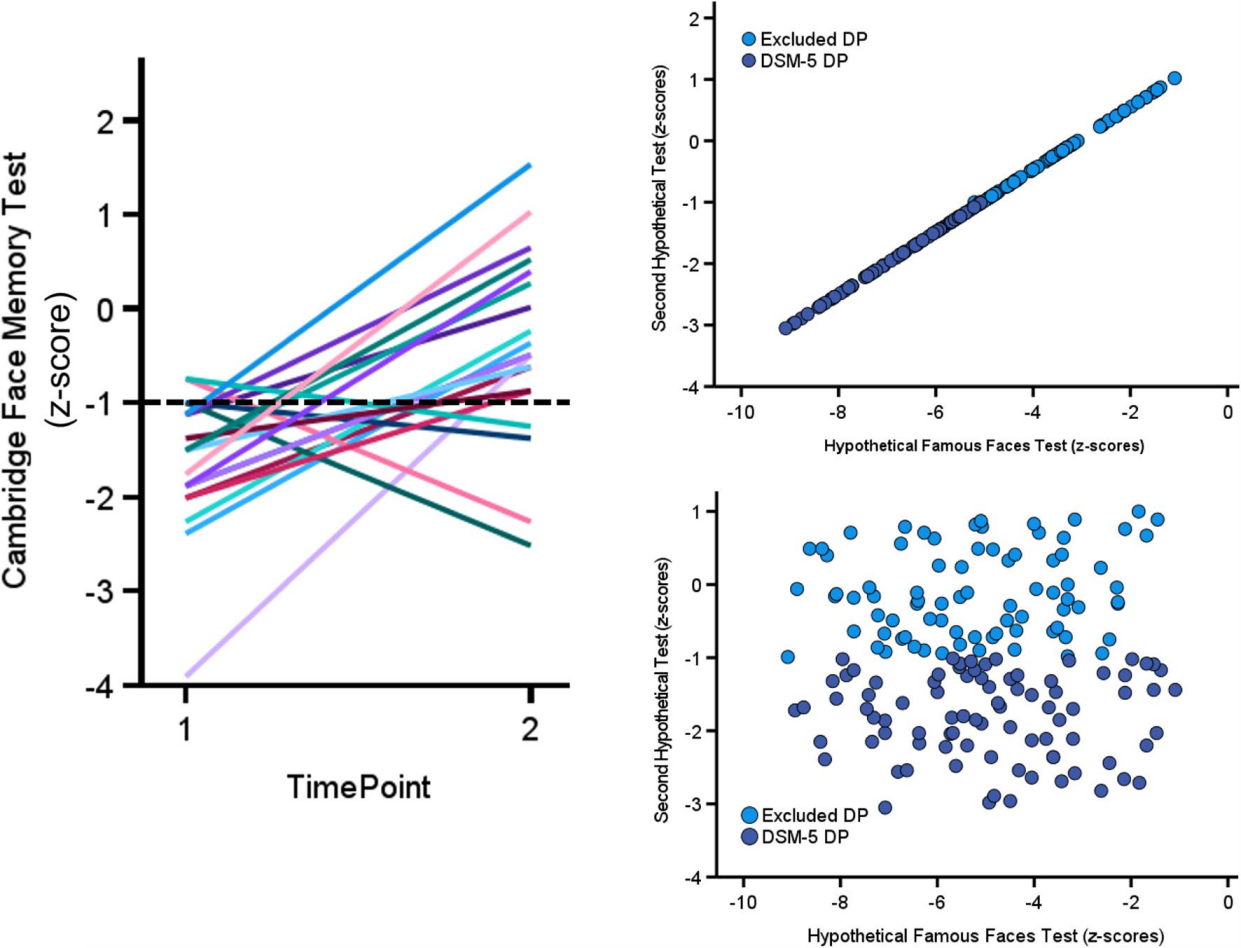
Top left panel illustrates how imperfect CFMT test-retest reliabilities will result in changes to potential diagnostic status (black dashed line represents -1 SD below neurotypical mean) from the first assessment timepoint to the second in 20 DP cases (Murray & Bate, 2020). Top right panel demonstrates how a perfect correlation (*r* = +1) between our two hypothetical diagnostic tests will result in only the most severely impaired FFT DP cases receiving a diagnosis. In the bottom right panel, where there is no correlation (*r* ≈ 0), the diagnosed cases will be sampled throughout the FFT distribution. Thus, the strength of the relationship between the two cognitive tests will produce unique biases in the types of cases we can diagnose and study. Please note, the FFT has 100% power and the second test roughly 50% in both plots on the right

Cognitive tests also suffer imperfect test–retest reliabilities too, whereby a patient can acquire a diagnosis one day, but then fail to gain one the next. To illustrate this, I reanalyzed data from Murray and Bate (2020) who retested DP cases days to months apart after an initial assessment on the CFMT. Out of their 70 cases, 29% failed to replicate their initial diagnostic status using the – 1 SD cutoff, shifting from DP to neurotypical, or from neurotypical to DP (Fig. 2). Importantly, the CFMT has been discoverable from Internet searches over the years, so those suspecting that they may have the condition could have taken it prior to formal testing. Given 80% of Murray and Bate’s (2020) cases that crossed the – 1 SD threshold on their second attempt moved from potentially diagnosed to missed should give cause for concern. This is because many DP cases will miss acquiring a diagnosis simply because of their curiosity to seek out an initial online CFMT self-assessment prior to contacting a clinician for testing. Thus, the DSM-5 will fail to diagnose many patients because of imperfect test–retest reliabilities^2^.

### Problems with two diagnostic tests

It is important to note that scoring below – 1 SD on a single cognitive test is not enough to acquire a diagnosis in the DSM-5 (Sachdev et al., 2014). A patient must score below – 1 SD on *two* tests (Sachdev et al., 2014). However, this additional requirement is especially problematic as it exacerbates the issue of missed diagnoses: maybe all cognitive tests suffer the validity and reliability issues outlined above. If true, our ability to diagnose any cognitive impairment will be constrained by the statistical power of the weakest test. Power in this case is simply the percentage of our patient sample that scores below any arbitrary cutoff (i.e., alpha) we have chosen for our two tests^3^ (McIntosh & Rittmo, 2021). When the Famous Faces Test requires a – 1 SD cutoff, analyzing data from recent papers suggests its power ranges from 65–85% (e.g., Bate, Bennetts, Gregory et al., 2019; Burns et al., 2023; Lowes et al., 2024). The CFMT is the most widely used standardized DP diagnostic assessment in combination with the FFT (Burns et al., 2023; DeGutis et al., 2023). However, it too misses many cases, exhibiting power of only 50–79% at the – 1 SD cutoff (e.g., Bate, Bennetts, Gregory et al., 2019; Burns et al., 2023; Lowes et al., 2024). If we require impairment on both the FFT and CFMT, then power will likely decline further, with it impossible to achieve overall power to diagnose higher than that provided by the lowest powered test.

To illustrate this, I plotted the FFT and CFMT data (Fig. 1) from a large sample of 165 self-identified DP cases reported in Bate, Bennetts, Gregory et al., 2019. Eighty-five percent met the – 1 SD diagnostic criteria on the Famous Faces Test, while 79% met criteria on the CFMT. When we required impairment on both tests, power fell below the lowest of the two, namely the CFMT, with 70% diagnosed. Many people who self-identify as having DP will therefore never acquire a diagnosis simply because of power constraints. Furthermore, modeling work has shown testing additional control participants will have limited scope for improving power (McIntosh & Rittmo, 2021).

Another issue with requiring impairment on two cognitive tests is that it misses cases who are, objectively speaking, highly abnormal in terms of their single test score. We can see this in the bottom left panel of Fig. 1. The FFT cut-off excludes cases who score more poorly than – 2 SDs on the CFMT, which is the two-tailed threshold for an individual’s test score being statistically significant in terms of atypicality. Despite these cases suffering severe difficulties when learning new faces, the DSM-5 requirement of impaired on two tests means they will never acquire a diagnosis. These problems appear even more pronounced when we examine cases excluded by the CFMT, with many scoring between – 2 and – 7 SDs on the FFT. Can we honestly state someone who scores almost – 7 SDs on the FFT does not have developmental prosopagnosia? I do not believe so. Especially when such cases may be spared in learning unfamiliar faces over several seconds, as tested by the CFMT, but fail to effectively recollect identity for long-term recognition, as is required in the FFT and daily life. Thus, while adding a DSM-5 diagnostic option of impaired on a single task at the – 2 SD level may counter these issues, the data shows the current DSM-5 excludes many objectively atypical DP cases.

Another problem with requiring two tests to diagnose neurocognitive disorders is that only one of them may have perfect, or close to perfect, validity. In DP, this would likely be some form of assessment that captures their failings to recognize personally familiar people in the real world. Even if this hypothetical task could detect impairment at the – 1 SD level in all cases, no other task would arguably be as good. This is because the defining problems of the condition are captured by this perfect test, and any other test will likely detect related, albeit imperfect, peripheral aspects of their day-to-day difficulties. As a result, requiring impairment on a second test will inevitably exclude patients from a diagnosis. This is demonstrated in a hypothetical Famous Faces Test (bottom right panel of Fig. 1) capable of detecting impairment in 100% of DP cases at the – 1 SD level. Despite this perfect assessment, overall power to diagnose a patient will be equal to that provided by the second test, namely the Cambridge Face Memory Test (79%), with many atypical FFT cases missed. Thus, if one cognitive task is perfect for diagnosing, then we should simply use one, rather than redundantly introducing a second as the DSM-5 recommends.

Relatedly, the DSM-5 only describes in general terms which two tests should be used to diagnose impairments within each of its six cognitive domains (DSM-5, 2014). For example, in the visual-motor category, face perception and/or recognition tasks are referred to as potential candidates with few details beyond that (DSM-5, 2014). This risks clinicians and researchers viewing perception (e.g., what’s makes this face unique?), unfamiliar face recognition (e.g., can I recognize a face that I have been briefly exposed to?), and familiar face recognition (e.g., can I identify a personally known or famous face?) tests as equally valid diagnostic tools. However, a Famous Faces Test that controls for familiarity is currently the best for detecting single case atypicality (e.g., Bate, Bennetts, Gregory et al., 2019), and the closest that can come to measuring the severity of symptoms described by those with DP (e.g., Bate, Bennetts, Gregory et al., 2019). Thus, if familiar face recognition is the cognitive construct that is impaired in this group, then we should only use diagnostic tasks that measure this construct. While perception and unfamiliar face recognition tasks may be useful for identifying subtypes of DP, they can lack the validity and sensitivity of the Famous Faces Test. Thus, the DSM-5 does not contain sufficient details that enable clinicians and researchers to make informed choices about which two diagnostic tasks are the most valid.

Another issue with the DSM-5 is that the strength of the relationship between the two tests will introduce a unique bias into the types of patients we can diagnose. This is illustrated in Fig. 2, with our 100% powered hypothetical FFT, and a second hypothetical test that only has around 50% power. When the correlation between the two is perfect (i.e., *r* = +1), we will exclusively diagnose the lowest scoring participants on the FFT test. By contrast, if there is no correlation between the two (i.e., *r* = 0), we will sample cases from throughout the FFT distribution. It is important to note that the further the relationship moves from *r* = +1 to *r* = 0, the probability of excluding cases who are more severely impaired on the FFT increases. Thus, while we are diagnosing a sample that reflects the breadth of the FFT distribution when *r* = 0, we will also exclude many highly atypical cases from a diagnosis too. The DSM-5 requirement of impaired on two tests will therefore introduce a correlation dependent bias in the types of patients we can ever diagnose, study, and treat.

In summary, the DSM-5 approach to diagnosing cognitive impairment is extremely limited due to issues in cognitive and behavioral testing. These include imperfect validities, test–retest reliabilities, and a failure to acknowledge diagnostic tests’ limitations. Similarly, requiring impairments on a second task will further exclude objectively atypical cases. By highlighting these pitfalls in DP, I encourage clinicians and researchers working in other neurocognitive disorders to reevaluate their diagnostic methods. Only by doing so can we hope to develop more sensitive, patient-centered, diagnostic approaches.

### Consequences of missed diagnoses

While it is important to acknowledge why missed diagnoses occur, it is equally important to recognize the myriad of negative consequences they create. For example, patients can question their own lived-in experiences and sanity after being told there is nothing wrong with them by a medical practitioner (Au et al., 2022; Eyal, 2022; Wise, 2022). This is a frequent occurrence in diverse conditions linked to problems in cognition, such as Long COVID (Au et al., 2022; Eyal, 2022), dementia (Nelson & O'Connor, 2008; Rentz et al., 2000; Rentz et al., 2004), electroconvulsive therapy patients (Rose, 2022; Rose et al., 2003), and DP (Burns et al., 2023). Without a formal diagnosis, patients will not be able to move forward with insurance claims, impacting their ability to acquire support and treatment. This will be particularly problematic if treatments are only effective at an early stage of disease, as those missed from a diagnosis will not be treated in time. Also, an absence of a diagnosis will block patients from legally protected, workplace-related, reasonable adjustments to accommodate their loss of function. This means that missed diagnoses will severely impact the lives of patients with neurocognitive disorders and their families.

The above could be described as patient focused issues, but missed diagnoses will also negatively impact science. For example, if only the most extremely impaired cases who score below – 1 SD are diagnosed, then prevalence rates and effect sizes of impairments will never be accurate, owing to missed cases being excluded from the top end of the disorder’s performance distribution. Such exclusions will undermine any epidemiological work that seeks to assess the etiology and outcomes of neurocognitive disorders, as missing cases will potentially bias results and waste vast resources.

**Fig. 3:**
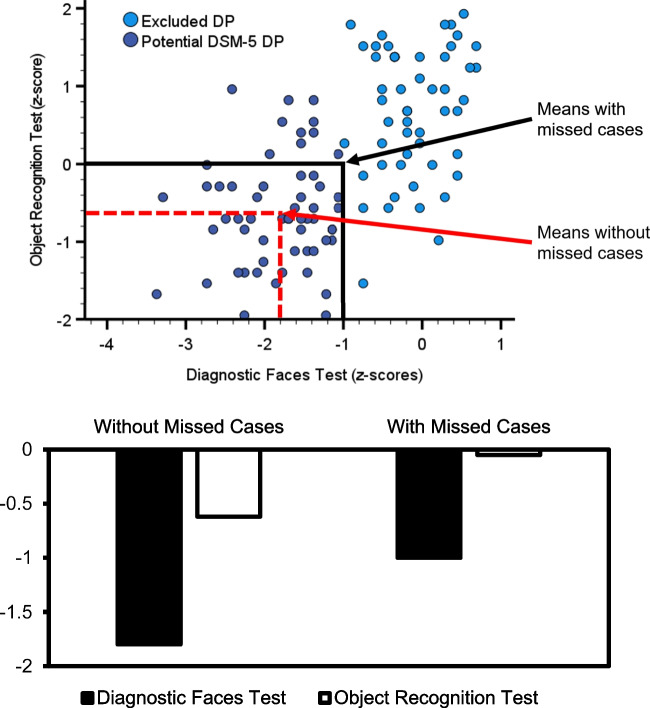
In the *top panel*, we present a hypothetical diagnostic faces test (*x*-axis) that is correlated with a hypothetical object recognition test (y-axis). The – 1 SD cutoff excludes roughly 50% of potential DP cases on this single test (*light blue circles*). When we plot the mean *z*-scores in the *bottom panel*, you can see that without the missed cases, DP is associated with face *and* object recognition impairments. This supports a cognitive model of shared underlying processes for the two abilities. However, when we include the DP cases who have been excluded from a potential DSM-5 diagnosis due to the – 1 SD cutoff, we find estimates of face recognition impairments in this group becoming milder, and object recognition deficits disappear. We now create a cognitive model that dissociates these two abilities. These issues will also be apparent in neurocognitive models if we use replace object recognition with neuroimaging measures, such as an fMRI BOLD response. Please note, I only used one diagnostic test here. These problems will likely become more pronounced with the introduction of a second diagnostic test as the DSM-5 requires

Missed cases will also impact neurocognitive models, because their absence risks altering dissociations and associations to such an extent, they render the model’s underlying evidence base meaningless. To illustrate this, imagine a hypothetical diagnostic test of face recognition has power of roughly 50% at the – 1 SD level, and is correlated with an object recognition test (Fig. 3). The effect size of impairment in potentially diagnosed DP cases on our faces test averages – 1.8 SDs below a neurotypical mean, with a comorbid impairment of – .62 SD in object recognition. These group level impairments suggest the two processes are to some extent not dissociable. Thus, a cognitive model derived from this data shows overlapping functionality between face and object recognition.

However, our 50% powered diagnostic test has excluded many DP cases. If we include them, then we will find we overestimated our DP group’s impairment on this task, as it is now reduced to – 1 SD. Importantly, the impairment in object recognition disappears. The resulting cognitive model now favors a dissociation between these processes^4^. Moreover, given object recognition is now intact, the correlation between faces and objects may be explained by another process. For example, attention is frequently cited as the domain general cause of such associations because face and object recognition are believed by many to be reliant upon dissociable brain networks (Kanwisher, 2017; Kanwisher & Yovel, 2006; McKone et al., 2007; although see Burns, Arnold et al. 2019; Gauthier, 2017) Thus, if attention were the cause of the relationship between face and object recognition, then adding data from an attention task as a covariate should abolish the link. Alternatively, the correlation could be due to domain general processes being utilized, but a dissociable face-specific component being impaired, hence the lack of group level object recognition impairments. Either way, exclusions similarly affect our cognitive model’s assumptions.

The same problem would also be apparent when identifying abnormalities in structural or functional MRI work. Imagine replacing object recognition in Fig. 3 with the neural activation in a brain region. This region’s BOLD response is correlated with performance on our hypothetical face recognition test used to diagnose DP. Again, as we excluded many of our DP cases, we find this brain region exhibits a lower-than-normal BOLD response,

suggesting it is atypical in DP. When we add the missed cases, this atypicality disappears. Our neural model therefore changes from this region seeming essential for face recognition, to being of limited importance and appearing intact in DP. Remarkably, a recent study showed DP cases exhibited reduced neural responsiveness across much of the brain (Jiahui et al., 2018). However, as these atypicalities were shown in only a minority of the most extremely impaired DP cases (i.e., diagnosed – 2 SDs on two face processing tests), they may as a population appear intact if we had included the full range of potential cases. Missed diagnoses will therefore impact virtually every area of cognitive and neurocognitive science, undermining any trust we have in the literature.

Failing to diagnose patients correctly also means treatments can erroneously appear effective due to statistical artefacts. This is because when we only diagnose and treat a sample that perform the lowest on cognitive assessments, post-treatment improvements identified through retests risk being nothing more than a regression to the mean (Barnett et al., 2005; Finney, 2008; Morton & Torgerson, 2005). That is, patients who score exceptionally poorly in the first instance to gain a diagnosis, will typically perform better on a retest due to the simple fact that there is only one direction their scores can go post treatment. Conversely, patients with the mildest impairments that fail to acquire a diagnosis may be the most responsive to interventions due to their residual cognitive abilities. Unfortunately, as they have been blocked from acquiring a diagnosis, they will never be included in clinical trials that assess a treatment’s efficacy. In summary, failed diagnoses will have a substantial, negative impact upon patients, their families, and science.

### Validating a symptom-based approach to diagnosing

Given the shortcomings of cognitive tests as diagnostic tools, we must explore viable alternatives when substantial overlaps exist between the performance distributions of neurotypicals and neurocognitive disorders, i.e., when the DSM-5 approach proves ineffective at distinguishing between impaired versus intact cognitive abilities at the level of the individual patient. One solution might be a symptom questionnaire if it were shown more effective at differentiating a patient’s complaints from neurotypicals. If so, we must validate it. One way of accomplishing this would be to pool missed cases’ cognitive task data together to enhance power for detecting objective impairments at the group level. This will transform missed patients' atypical levels of symptoms from mere subjective complaints, into validated indices of underlying cognitive deficits. Moreover, the remaining patients’ self-reported complaints will have been validated at the – 1 SD level on two cognitive tasks via the DSM-5 approach. Given these dual approaches to detecting impairment across the whole patient sample, a symptom-based method, if more effective, would become a viable diagnostic alternative to the DSM-5.

As a proof of concept, I assessed a large sample (*n* = 61) of self-identified developmental prosopagnosia cases. I first quantified the proportion of these individuals that would fail to acquire a diagnosis through the DSM-5, then sought to identify their group-level impairments on multiple tasks to validate a symptom-based approach to diagnosing. Finally, I replicated these deficits in a separately collected sample (Bate, Bennetts, Gregory et al., 2019), before assessing all data using meta-analyses. If missed cases exhibit deficits in face processing, it will confirm the DSM-5 fails to diagnose objectively impaired DP. This would mean the one-size-fits-all approach recommended by the DSM-5 does not always work, as it blocks people with objective impairments from acquiring a diagnosis. Instead, we should tailor diagnostic approaches to the specific cognitive impairments we are assessing.

## Methods

### Transparency and openness

All data required to replicate our results is available on the Open Science Framework (https://osf.io/3x86n/). As I do not own the copyright for the tasks, I do not make them available. The PI20 is freely available in the original paper (Shah et al., 2015).

### Participants

I report how I determined my sample size, all data exclusions, all manipulations, and all measures in the study. The first DP sample comprised 62 cases whose ages ranged between 18 and 72 years old (*M* = 41.5, *SD* = 14), with nine males, and three who identified as neither male nor female. All cases reported severe, lifelong troubles with faces with no obvious historical reason for it being acquired (Burns et al., 2023). Due to COVID-19 restrictions, all tests were carried out online. One DP case was excluded for failing two out of two attention checks throughout the tasks. Another failed to move any faces during the Cambridge Face Perception Test: i.e., 62-year-old female who made 96 errors on upright and inverted (Table 1). Presumably, she had a problem with her mouse on this task, so included her other data where possible. This left 61 DP cases for all assessments, except for the CFPT-related measures (*n* = 60). Data was not collected on participants’ other cultural backgrounds (e.g., socioeconomic status; Appelbaum et al., 2018).

**Table 1:** Percentage of DSM-5 missed DP cases (*top row*) and their mean impairment *z*-scores from the current sample and Bate, Bennetts, Gregory et al. (2019). While my DSM-5 missed cases were impaired on all measures, Bate, Bennetts, Gregory et al. (2019) cases were only impaired on CFMT and FFT. Please note, Bate, Bennetts, Gregory et al. (2019) DPs did not complete the PI20 or inverted portion of the CFPT. *Asterisks* indicate significant impairments relative to neurotypical controls at < .05, SDs are in brackets

DSM-5 missed cases	Current sample	Bate, Bennetts, Gregory et al. (2019)
% of total DPs	38%	30%
CFPT upright	– .6* (.85)	– .18 (.91)
CFPT holistic	– .52* (.52)	N/A
CFMT	– .83* (.86)	– .82* (.94)
FFT	– .45* (.77)	– 1.58* (1.91)
PI20	– 4.45* (.91)	N/A

Fifty-two controls participated: ages ranged 22–68 years old (*M* = 38.8, *SD* = 11.3) with 31 males. While there were many more males than females in this group, and women typically recognize faces better than men (Herlitz & Lovén, 2013), there is a concern this may result in an underestimation of face recognition difficulties in our DP sample. I ran independent *t* tests on the control data from all measures and found no significant gender-related effects [all *p*s > .08, BF10 ≤ 1], thus do not include gender in any of my analyses.

Three control males reported lifelong troubles with faces, and scored highly atypical (i.e., significant at the one-tailed alpha threshold) on the prosopagnosia index questionnaire (74, 68, and 63), suggesting they likely have DP. I therefore excluded them from all analyses.

Power was difficult to estimate a priori, as it was unclear just how many cases would fail to acquire a diagnosis using the DSM-5 approach, or what their level of impairment might be. Given the DSM-5 diagnostic criterion is – 1 SD on two cognitive tests, and the average level of cognitive impairment in the self-identified DP cases is just below – 2 SDs on the CFMT (Burns et al., 2023), I presumed the group-based deficits in the DSM-5 missed cases would be close to – 1 SD. This is because their distribution was potentially skewed (i.e., being the high-performing tail-end of the normally distributed DP group), with most cases congregating around this level. An effect size (Cohen’s* d* = .8) was chosen based on this hypothesis. This required 26 participants in the excluded DP group (i.e., those who failed to meet DSM-5 criteria) and 26 in the control group, to detect effects with an alpha of .05 and power of 80%. A post hoc power analysis based on the 23 DP cases who were missed by the DSM-5 approach, and 48 controls, suggested that power was 87%, with 80% power to detect down to Cohen’s* d* = .72.

To avoid reducing power further, I do not make any corrections for multiple comparisons, especially given these are difficult to recruit cases. However, to reassure readers the results were not false positives, I largely replicated my findings in an additional DP group (Bate, Bennetts, Gregory et al., 2019). Moreover, I ran *p* curve meta-analyses to ensure any impairments detected in cases excluded from a DSM-5 diagnosis had evidential value (Simonsohn et al., 2014).

None of our experiments or hypotheses were formally pre-registered. While low-level object recognition difficulties were not tested (e.g., Birmingham Object Recognition Battery: BORB; Riddoch & Humphreys, 2022), no DP cases disclosed general problems with vision, and when I historically included the BORB in DP testing, those with the condition did not exhibit problems. This has been objectively confirmed across 200 cases in recent papers (Bate, Bennetts, Gregory et al., 2019; Lowes et al., 2024), with only one DP case exhibiting possible difficulty (Lowes et al., 2024). Thus, despite some concerns about omitting such testing (Nørkær et al., 2024), low-level vision problems are no more prevalent in DP than neurotypicals, meaning that such tasks are unnecessarily onerous for DP testing in the absence of patient complaints.

### Procedure and materials

A battery of cognitive tests that have historically been used to diagnose developmental prosopagnosia (Burns et al., 2023; DeGutis et al., 2023) were completed by all participants. These included a 72-trial assessment of unfamiliar face memory (the Cambridge Face Memory Test: CFMT; Duchaine & Nakayama, 2006), an unfamiliar face perception test which included eight upright trials and eight inverted (the Cambridge Face Perception Test: CFPT; Duchaine, Germine et al. 2007, Duchaine, Yovel et al. 2007), and a validated, 30-trial familiar face memory test (i.e., Famous Faces Test: FFT) developed by my own lab (Burns et al., 2023). FFT scores were not corrected for participants’ familiarity (i.e., we did not provide celebrities’ names afterwards to adjust participants’ possible scores based on these responses). Participants also completed the Prosopagnosia Index questionnaire (Shah et al., 2015). All DP cases’ individual scores and full test details are presented in Supplementary Information here (https://osf.io/3x86n/).

The Bate, Bennetts, Gregory et al. (2019) sample completed their own FFT, the CFMT and only the eight CFPT upright trials. Bate, Bennetts, Gregory et al. (2019) FFT differed from mine in a few ways. First, they used two versions, one for participants aged under 35 years old, and one for participants aged ≥ 35; faces were selected based on pilot work with these age groups. Both versions used 60 faces, and in contrast to my FFT, each participant’s total possible correct scores were adjusted by removing celebrities they were not familiar with by name. Full details of Bate, Bennetts, Gregory et al. (2019) methods can be found in their open access paper.

Ethical approval was granted by Edge Hill University Ethics Review Board, with all work carried out in accordance with the 1964 Helsinki Declaration on human testing. All participants gave informed consent, and for their anonymized data to be published.

## Results

### The DSM-5 diagnoses 62% of DP cases, the symptom-based approach 100%

The DSM-5 approach to neurocognitive disorders requires participants to score more poorly than – 1 SD from the neurotypical mean on two cognitive tests. DeGutis et al. (2023) recommended that these should be two tests of face memory when diagnosing DP. I used this guidance with the CFMT and FFT because they have historically been the two most widely used tests to diagnose DP (Burns et al., 2023; DeGutis et al., 2023). This revealed a striking 38% of self-identified DP cases were excluded from a diagnosis. By contrast, 100% were classified as atypical using the prosopagnosia index via a Crawford’s *t* test (Crawford & Howell, 1998).

**Fig. 4:**
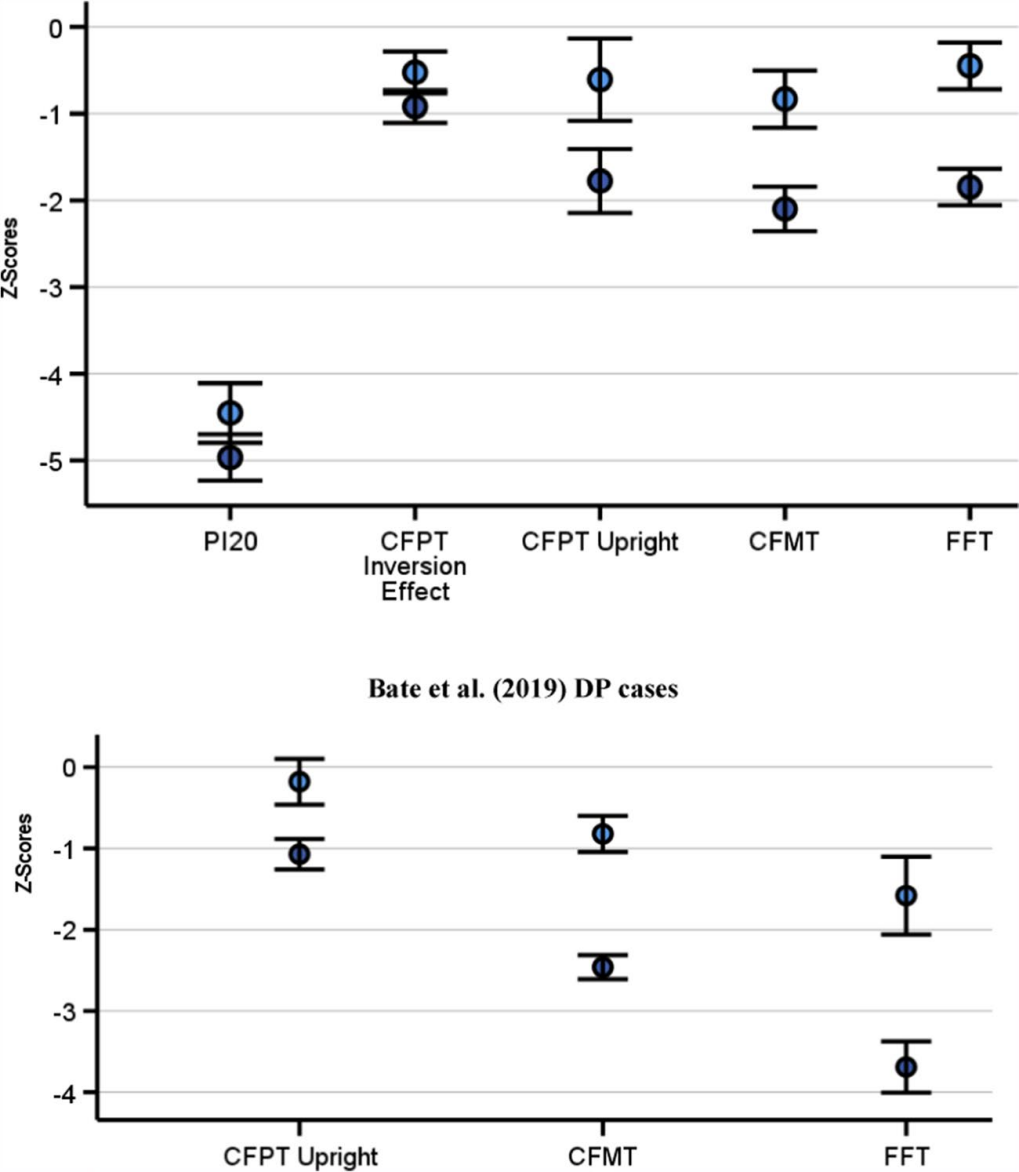
DSM-5 missed DP cases are objectively impaired on multiple cognitive measures. In the *top panel*, DP cases excluded from a DSM-5 diagnosis (*light blue circles*) exhibited highly atypical prosopagnosia symptoms (i.e., PI20) and objective impairments on every cognitive task related measure. These deficits validate their subjective complaints. Please note, the DSM-5 DP group’s mean *z*-scores are plotted for context (*dark blue*): all were significantly milder on all measures [all *p*s < .022]. In the *bottom panel*, the excluded DP cases from Bate, Bennetts, Gregory et al. (2019) were impaired on the CFMT and FFT. While the CFPT upright *z*-scores were not significantly different from controls, they were in the same direction (i.e., impaired) as my DP sample. Please note, Bate, Bennetts, Gregory et al. (2019) FFT impairments were likely much larger than my sample because the former controlled for participants’ familiarity with the faces, while I did not

We wanted to assess whether the DSM-5 approach diagnosed DP cases who reported more severe symptoms than those that were excluded. To ensure higher power to detect potential symptom differences between the groups, and because all DP cases were highly abnormal in their self-reported symptoms at the individual level (i.e., more than – 2 SDs below a neurotypical mean), I performed a between participants *t* test on the DSM-5 diagnosed and excluded DP cases’ PI20 scores, i.e., I did not include control data. This revealed excluded cases reported fewer problems with faces [*M* = 79, *SD* = 8.17] than those who acquired a diagnosis using the DSM-5 approach [*M* = 83.61, *SD* = 6.84, *t*(59) = 2.37, *p* = .021, Cohen’s *d* = .63]. The DSM-5 approach therefore seems to capture some of the symptom differences between those who are diagnosed and those that are not. Figure 4 illustrates the mean level of *z*-score impairment for both the DP groups across all measures.

### Excluded DP cases are impaired in unfamiliar face memory

To validate excluded cases’ highly abnormal symptom complaints, we compared their CFMT scores to the neurotypical group (Fig. 4). This confirmed they were impaired in unfamiliar face memory abilities [Excluded DP *M* = 51.52 trials correct, *SD* = 8.39; Neurotypical *M* = 59.7, *SD* = 9.8, *t*(69) = 3.43, *p* < .001, Cohen’s* d* = .87]. Thus, the DSM-5 approach to diagnosing neurocognitive disorders excludes DP cases who exhibit objective difficulties with unfamiliar faces.

### Excluded DP cases are impaired in upright face perception

The Cambridge Face Perception Test (Duchaine, Germine & Nakayama, 2007) is often used in neuropsychological assessment to identify DP cases who suffer from the apperceptive form of the disorder (Biotti & Cook, 2018), i.e., those that have difficulties telling faces apart from one another. I used the CFPT to assess whether the DSM-5 approach would exclude DP cases who were objectively impaired in face perception. Confirming this, they made significantly more errors on this task [*M* = 41.9 errors, *SD* = 12.02] than the control group [*M* = 33.33, *SD* = 14.16, *t*(69) = 2.5, *p* = .015, Cohen’s* d* = .64]. This means we have validated excluded cases’ self-reported complaints in unfamiliar face memory and face perception.

### Excluded DP cases’ holistic perception abilities are impaired

Holistic perception is characterized by the brain encoding an interaction between a face’s features to create a salient, unitary percept (Burns & Wilcockson, 2019; Dal Lago et al., 2023, 2024; Luo et al., 2017; Maw et al., 2023), with reduced abilities a suggested cause of face processing difficulties in DP (Avidan et al., 2011; DeGutis et al., 2012; although see: Biotti et al., 2019). I assessed whether excluded DP cases were impaired in holistic perception using the corrected inversion scores from the CFPT as an index (Supplementary Information). This revealed that excluded cases [*M* = .69, *SD* = .53] exhibited smaller inversion effects in comparison to our controls [*M* = 1.16, *SD* = .9, *t*(66.04) = 2.74, *p* = .008, Cohen’s* d* = .58]. We have therefore validated excluded DP cases’ symptom complaints via three objective measures: CFMT, CFPT upright and CFPT Holistic Perception.

### Excluded DP cases exhibit impairments when judging famous faces

DSM-5 excluded DP cases exhibited deficits in face perception and unfamiliar face recognition. However, familiar faces, such as friends and celebrities, are thought processed in a partially distinct way from unfamiliar faces (Ellis et al., 1979; Johnston & Edmonds, 2009; Megreya & Burton, 2006). I therefore assessed the presence of familiar face impairments in excluded cases using famous faces. This confirmed those who failed to acquire a diagnosis through the DSM-5 [*M* = 17.87 trials correct, *SD* = 4.75] identified fewer celebrity faces in comparison to controls [*M* = 20.65 correct, *SD* = 6.2, *t*(55.34) = 2.08, *p* = .042, Cohen’s* d* = .48]. This means excluded DP cases exhibited objective impairments on all four measures of face processing.

### Replication: DSM-5 excluded DP cases’ exhibit face memory impairments

Bate, Bennetts, Gregory et al. (2019) used the CFPT upright, CFMT and their own FFT to test 165 self-identified DP cases. Strikingly, 30% of these individuals failed to meet the DSM-5 criteria (Table 1). While slightly lower than the 38% I found with my sample, it largely replicated this figure, i.e., a substantial number of potential DP cases will fail to acquire a diagnosis using the DSM-5 method. Table 1 shows the mean *z*-scores for my data and Bate, Bennetts, Gregory et al. (2019) side by side for easy comparison. Importantly, analyses of Bate, Bennetts, Gregory et al. (2019) replicated my finding of missed cases exhibiting significant unfamiliar and familiar face memory impairments [*p*s < .013], but did not corroborate the upright CFPT difficulties [*p* = .24].

### Meta-analysis support the existence of objective impairments in missed cases

It is increasingly common for researchers to assess multi-experiment evidence presented within their papers through a meta-analysis (e.g., Alves et al., 2017; Van Kuijk et al., 2018). This is an important antidote to the replication crisis psychology has faced in recent years, as it can provide support that any studied effect is real (Simonsohn et al., 2014). The *p* curve is a widely used (Simonsohn et al., 2014) and effective (Lakens, 2023) meta-analysis technique that only uses significant *p* values. It is based on the fact that when true effects exist, *p* values will exhibit a right skew when plotted together; with the highest frequency of values congregating around .01 (Burns & Bukach, 2023; Simonsohn et al., 2014). By contrast, if the null hypothesis is true, then *p* values should appear flat (i.e., uniformly distributed).

**Fig. 5:**
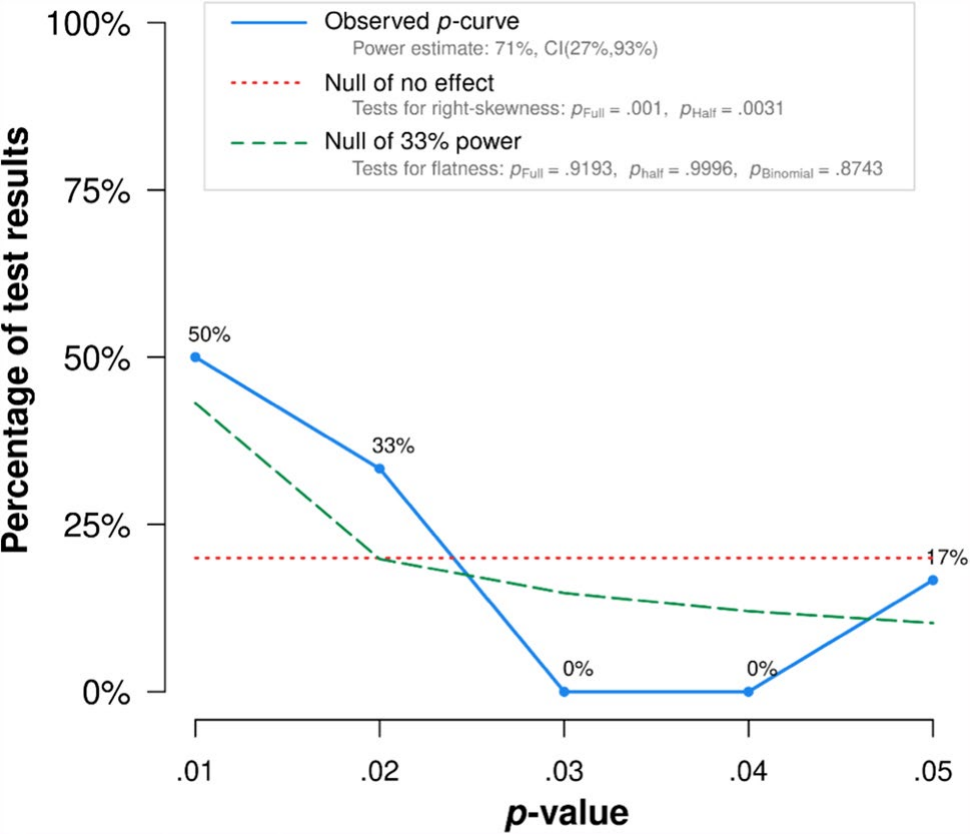
Meta-analyses support cognitive impairments in excluded DP cases. All *p* values in support of missed cases’ objective cognitive impairments from my sample and Bate, Bennetts, Gregory et al. (2019) exhibit a right-skew distribution, with the *p* curve analysis statistically significant. This means that missed cases’ complaints are validated by objective impairments in face processing

I performed a *p* curve using all significant *p* values that confirmed missed cases’ objective cognitive impairments from mine and the Bate, Bennetts, Gregory et al. (2019) samples. This meta-analysis was significant [full curve: *Z* = – 3.08, *p* = .001; half curve: *Z* = – 2.74, *p* = .003], thus supporting the proposal that DSM-5 missed cases’ cognitive impairments contain evidential value. Figure 5 illustrates the right-skewed distribution of *p* values indicating they reflect a real effect. As the holistic perception-related *p* value may not be entirely independent from the CFPT upright *p* value, I ran the analysis again with the former excluded: the *p* curve remained significant [full curve: *Z* = – 2.92, *p* < .002; half curve: Z = – 2.83, *p* < .003]. This was replicated when I replaced the CFPT upright *p* value with the CFPT holistic perception *p* value [full curve: *Z* = – 3.13, *p* < .001; half curve: *Z* = – 3.18, *p* < .001]. In summary, meta-analyses confirm DSM-5 missed cases’ objective impairments in face processing.

## Discussion

**Fig. 6:**
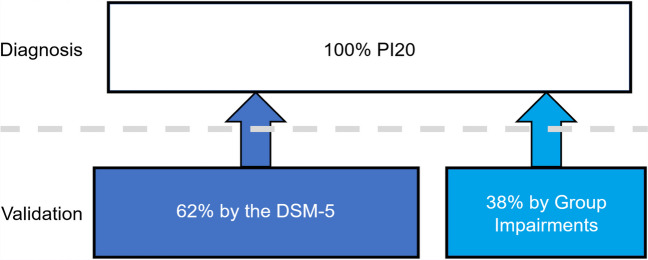
How the symptom-based approach to diagnosing is validated by objective impairments in DP. The PI20 diagnoses 100% of DP cases as atypical in their symptoms (*top*). Sixty-two percent of these self-identified DP cases were validated as objectively impaired at the – 1 SD level on the FFT and CFMT, with the remaining 38% validated through deficits at the group level in their FFT, CFMT, CFPT upright and holistic perception measures. Thus, when a DP case reports atypical symptoms via the prosopagnosia index questionnaire, we can be confident that they reflect underlying cognitive impairments

The DSM-5 approach to diagnosing cognitive impairment requires a patient to score beyond – 1 SD on two objective tests of behavior or cognition (Sachdev et al., 2014). I outlined how this approach will result in missed diagnoses, using developmental prosopagnosia as a proof of concept. Testing two large, independently collected samples, I showed the DSM-5 excluded between 30 and 38% self-identified DP cases. Importantly, both groups of missed cases exhibited impairments in familiar and unfamiliar face memory, with one sample displaying face perception difficulties too^5^. These problems were further confirmed through meta-analyses. The prosopagnosia index questionnaire proved more effective as a diagnostic tool than the DSM-5, with the former identifying significant atypicality in 100% of cases at the individual level, in contrast to the latter that ranged between 62 and 70%. It is important to recognize that a diagnosis of DP based on a case’s symptoms is no longer a purely subjective measure, but reflects multiple, underlying objective impairments. These deficits were found using the DSM-5 approach for individual cases at the – 1 SD threshold on two tests, or when that failed, at the level of the group in excluded DP, thus validating the prosopagnosia index as a diagnostic tool (Fig. 6).

This study is an important demonstration of how cognitive tests can fail to diagnose a substantial proportion of objectively impaired patients. While I have shown this in one clinically relevant population, it is frequent to find substantial overlaps in the performance distributions of those who report cognitive impairments in other disorders versus those who are neurotypical (Beishon et al., 2019; Burns et al., 2023; Costas-Carrera et al., 2022; Eyal, 2022; Nelson & O'Connor, 2008; Potts et al., 2022; Rentz et al., 2000, 2004). This should pose as a warning to any clinician or researcher who tries to rigidly enforce a diagnostic criterion, such as the DSM-5 cutoff of – 1 SD on two cognitive tests, when it clearly does not fit the patient population. Instead, I advocate the use of a transdiagnostic, data-driven approach, in which the whole range of the possible patient sample self-reporting complaints are tested (Astle et al., 2022; Burns et al., 2023; Epihova & Astle, 2024), to identify, and correct, deficiencies in diagnostic tests and cutoffs. Only by doing so, and including them in our work, can we hope to improve our methods (Burns et al., 2023; Epihova & Astle, 2024).

If a symptom-based approach is more effective at identifying a patient’s atypicality in daily life, then I recommend validating it, and using it, to replace the cognitive or behavioral test-based approach proposed by the DSM-5. We can validate a symptom questionnaire, such as the prosopagnosia index, by identifying group based, objective impairments in missed cases on multiple tasks. By contrast, the remaining DP cases are already validated via the DSM-5 approach at the level of the individual patient. One benefit of using a symptom-based approach to diagnosing DP is that it may not be susceptible to the biases that face recognition tests suffer from. For example, women are typically better at identifying still images of faces in cognitive tests than men (Herlitz & Lovén, 2013; Wright & Sladden, 2003), and people are typically better at recognizing faces of their own ethnicity (Bate, Bennetts, Hasshim et al., 2019; Burns, Tree et al., 2019; Childs et al., 2021; Estudillo et al., 2020; Meissner & Brigham, 2001), and ages (Rhodes & Anastasi, 2012). Given that standardized cognitive tests (e.g., CFMT, CFPT) almost always contain images of young adults, they will underestimate face processing abilities of participants furthest away from these age groups (Burns, 2023). While some may have concerns that a symptom-based approach is susceptible to malingering patients (i.e., they can be easily faked), these issues are equally true of cognitive tests (Suhr et al., 2008, 2021). Moreover, an argument could be made that DP cases failing to meet DSM-5 criteria are highly unlikely to be malingerers, given the fact that they perform *above* diagnostic cutoffs on cognitive tasks, i.e., malingerers would likely exaggerate problems on the Famous and Cambridge Face Memory Tests.

Of course, it is theoretically possible a single cognitive test may detect atypicality in every patient. Or maybe when using two tests, at least one will always detect a patient’s cognitive impairment at the – 2 SD level. In such cases, I would recommend loosening the DSM-5 criteria. For example, if we added an additional option to diagnose based on a single test (i.e., – 2 SD cutoff), then we would remedy the DSM-5’s problems in missing highly atypical cases. Similarly, incorporating patients’ response times, in contrast to the historical reliance on accuracy rates, improves cognitive tests’ diagnostic sensitivity (Lowes et al., 2024). Thus, small modifications can result in improvements to the current DSM-5 method. Simply put, I would recommend using the most effective test for providing a diagnosis. This would prove useful, in contrast to the self-reported symptom approach, when patients are lacking awareness of their cognitive difficulties. The important message here for readers is that not every cognitive impairment will be easily diagnosed by the one-size-fits-all approach endorsed by the DSM-5, i.e., impaired at – 1 SD on two tests. When you consider the heterogeneity of patients’ impairments, and the heterogeneity in cognitive tests’ validities and reliabilities, it seems implausible that such a rigid method will work ubiquitously. We must tailor how impairments are diagnosed by assessing our best options within patient samples. Only by using bespoke, rather than general, approaches can we improve patient support and science.

I should add that there is some merit in the DSM-5 method. While I reject it as a diagnostic approach in the context of DP, it did distinguish between self-reported symptoms in those it diagnosed, versus those that it missed. This shows the DSM-5 can reflect cases’ differing levels of symptoms. There is a great deal of interest in the face recognition literature as to whether people have insights into their cognitive abilities (Bobak et al., 2019; Estudillo & Wong, 2021; Gehdu et al., 2023; Gray et al., 2017; Livingston & Shah, 2018; Matsuyoshi & Watanabe, 2021; Nørkær et al., 2023; Oishi et al., 2024; Palermo et al., 2017; Shah et al., 2015; Ventura et al., 2018). I have shown those with developmental prosopagnosia exhibit accurate insights into the existence of their objective impairments, and their severity, given the graded symptom levels between the DP cases on either side of the DSM-5 cutoff. This rejects suggestions that excluded cases are misinterpreting their face recognition abilities, or that they are suffering from a failure of meta-cognition (Arizpe et al., 2019; De Haan, 1999; DeGutis et al., 2023).

Another benefit of the symptom-based approach is that it is much shorter (i.e., a couple minutes) than the battery of cognitive tests we typically ask DP cases to complete (e.g., at least 40–60 min). This means a symptom questionnaire, once validated as a diagnostic tool, can save patients and clinicians valuable time in clinical settings. Also, there are no standardized cognitive assessments for DP in all ethnicities as cognitive tests used to diagnose DP are frequently geared towards Caucasian samples (e.g., Duchaine & Nakayama, 2006; Duchaine, Yovel et al., 2007). This makes them exclusionary given there are substantial performance variations when recognizing faces from other ethnicities, e.g., Caucasians will often exhibit problems recognizing Asian faces (e.g., Bate, Bennetts, Hasshim et al., 2019; Childs et al., 2021; Meissner & Brigham, 2001). A symptom-based approach should, in theory, negate these issues to some extent.

It is important to note some believe symptoms alone should never be used when diagnosing cognitive impairment (DeGutis et al., 2023; DeHaan, 1999; Nørkær et al., 2024), and that the solution to missed diagnoses is to develop more sensitive experimental tests (DeGutis et al., 2023). I have shown here that pooling missed cases’ data reveals group level impairments that are not otherwise detectable using the DSM-5. Thus, when a patient is diagnosed through the symptom-based approach, we can be confident their atypical symptoms reflect underlying objective impairments on multiple experimental measures (Fig. 6). I agree cognitive tests need to be improved in their sensitivity, validity, and reliability, but such improvements will further validate the symptom-based approach. Imagine we develop a cognitive testing battery that matches the PI20’s sensitivity, i.e., identifying all cases as atypical at the – 2 SD level. Why would we use such time-consuming tasks in overstretched clinical practices when we have a validated, and rapid, symptom questionnaire at our disposal? If we must wait for cognitive tests to improve, we will only perpetuate the problems outlined in the Introduction, and block 30–38% of objectively impaired developmental prosopagnosia cases from a diagnosis. Without the symptom-based approach, such individuals will be unable to access essential treatments, support, and legal protections in the workplace.

I must acknowledge there may be limitations to a symptom-based approach. For example, prior work has shown, albeit not with the prosopagnosia index, that symptom questionnaires can be susceptible to pathologizing normal behaviors in one culture over the other (Norbury & Sparks, 2013). However, given the vast numbers of cases missed when using cognitive tests in DP, potential cross-cultural issues in symptoms are, in my opinion, likely to have a much smaller impact in terms of missed diagnoses. Also, the PI20 (Sun et al., 2021) outperforms the CFMT (Murray & Bate, 2020; Wilmer et al., 2010) in test–retest reliabilities, meaning that a patient’s diagnostic status is less susceptible to changing from one day to the next, in contrast to a symptom-based approach. Despite this, it is recommended that a team independent of the scale developers assess such questionnaires, to remove potentially redundant items (Boateng et al., 2018). While this has been done to some extent with the PI20, the new scale was designed to improve the detection of neurotypical face recognition abilities, not developmental prosopagnosia symptoms (Bobak et al., 2019). This means there may be some benefits from further PI20 refinement. However, in our sample of 61 DP cases, it performed exceptionally well, identifying 100% of cases as suffering atypical levels of prosopagnosia symptoms beyond the neurotypical – 2 SD cutoff, i.e., no self-identified DP cases were erroneously reporting symptoms in the neurotypical range.

In summary, I have shed light on the limitations of the DSM-5 approach to diagnosing neurocognitive disorders, using developmental prosopagnosia as a compelling case in point. The conventional DSM-5 criterion of scoring below – 1 SD on two objective tasks excludes a significant percentage (i.e., 30–38%) of individuals who report severe problems in daily life. By introducing a symptom-based approach, we have identified excluded DP cases’ complaints as significantly atypical in all instances, and validated them through their underlying objective impairments. This offers a more comprehensive and patient-centered perspective on diagnoses, acknowledging the limitations of cognitive tests. These findings, although focused on developmental prosopagnosia, serve as a crucial reminder to clinicians and researchers that diagnostic criteria must be tailored to the unique characteristics of the patient population. Embracing a data-driven approach through such cases’ suspected issues can lead to a more effective diagnostic method and improve the accuracy of assessments. If a symptom-based approach demonstrates superior effectiveness in identifying atypicality in daily life, as we have shown here, then it should replace the DSM-5 method. By doing so, we can enhance the diagnostic process, making it more inclusive, unbiased, and ultimately, more reflective of real-world cognitive functioning.

## Data Availability

The data required to replicate my Results is available on the Open Science Framework (https://osf.io/3x86n/)

## Footnotes

1 Curiously, video clips of famous faces abolish the recognition impairments that are apparent when still images are used (Bennetts et al., 2015). Does normal performance on what is a more ecologically valid test due to the inclusion of movement, not suggest that cognitive tests fail to capture something with regards to DP cases’ complaints? Maybe diagnosing DP cases through still image recognition impairments, we are removing those who are largely intact on this task, but who may suffer difficulties integrating identity related information when faces are moving. Anecdotally, DP cases who perform too well on cognitive tests to acquire a diagnosis have told me that they find moving faces particularly challenging in contrast to photographs.

2 Please note, Murray and Bate (2020) showed changing diagnostic status was also an issue when using the – 2 SD cutoff.

3 My discussion of power limitations is comparatively brief compared to prior works on the subject in neuropsychology (McIntosh & Rittmo, 2021) and DP (Burns et al., 2023). I recommend both papers, but especially the first which provides a convincing demonstration of how cognitive tests can lack power.

4 Please note, excluded cases will only affect neurocognitive models if there is a relationship between face and object recognition. If there is no correlation (i.e., *r* = 0), then missed diagnoses will only alter the severity of group level face processing impairments in DP. By contrast, object recognition will not be affected.

5 There are a few potential reasons why the excluded DP cases from Bate, Bennetts, Gregory et al. (2019) failed to exhibit perceptual impairments in contrast to my own: their DP group was several years older and contained proportionally fewer women, they excluded cases who were high in autistic traits, and they corrected famous face scores for familiarity which would have changed the types of cases diagnosed. Any, or all, of these differences may have contributed to the absence of perceptual impairments in the Bate, Bennetts, Gregory et al. (2019) sample, although the direction of effect, while much smaller, was the same.

## Code availability

I do not own the copyright for many of the tests used so do not make them available, but I thank Brad Duchaine for providing the CFMT and CFPT. The PI20 can be found in the paper it was first reported on (Shah et al., 2015).
